# Acute post-traumatic encephalocele in a child: CT and MRI features

**DOI:** 10.1259/bjrcr.20150170

**Published:** 2016-11-02

**Authors:** Abhinav Aggarwal, Ashish Kumar Gupta, Aakriti Kapoor Aggarwal

**Affiliations:** ^1^Department of Radiodiagnosis, Rajiv Gandhi Cancer Institute & Research Centre, Rohini, New Delhi, India; ^2^Department of Radiodiagnosis, Shri Ram Murti Smarak Institute of Medical Sciences, Bareilly, India; ^3^Department of Radiodiagnosis, Medanta - The Medicity, Gurgaon, Haryana, India

## Abstract

Orbital trauma is a commonly encountered entity in clinical practice, especially in cases of head trauma. Although fractures of the orbit are rare, they can present in an emergency setting owing to associated complications such as orbital encephaloceles. We present a case of a paediatric male patient who presented with post-traumatic proptosis and diminution of vision and was diagnosed with orbital encephalocele. The child's vision recovered completely and proptosis of the eye decreased, compared with the time of presentation. Despite persistent counselling, the patient and his parents did not give consent for surgical correction and he was discharged as leaving against medical advice. Post-traumatic orbital encephaloceles are caused as a result of blunt cranial trauma. Early treatment and diagnosis is extremely important in the optimum management and good long-term prognosis of the patient.

## Summary

Post-traumatic fractures of the roof of the orbit are rare.^1^ These are seldom related with orbital blepharocele or encephalocele.^2,3^ Very few cases of orbital encephalocele have been reported, with a study published in 2010 stating the number such cases to be 25.^4^ The first case was reported in 1951.^5^ Orbital encephalocele is a result of frontal impact or lateral blow to the orbit.^3^ A major complication of orbital encephalocele is diminution of vision owing to compression of the optic nerve, which is particularly increased by the orbital walls forming a closed cavity. The most common symptoms include proptosis and visual disturbances. Early diagnosis of orbital encepaloceles is extremely important and cross-sectional imaging plays an important role. We present a case of a 12-year-old male child who developed unilateral post-traumatic orbital encephalocele.

## Clinical presentation

A 12-year-old male presented to the neurosurgery department of our hospital in a semiconscious condition with complaints of vomiting, proptosis, blackening of upper eyelid and significant diminution of vision of the right eye with an evolution of 6 h. The patient reported falling from a height (trauma) 6 h earlier. The patient had a Glasgow Coma Scale score of 10/15 (E2M5V3). The patient also had swelling of the scalp over the right frontal region. There was no history of nasal bleed or loss of consciousness. On clinical examination, periorbital oedema was noted. A provisional diagnosis of orbital haematoma was made in accordance with the history of the patient and a non-contrast-enhanced CT scan of the head and orbit was advised.

## Differential diagnosis

Based on the patient's history and clinical examination, our first diagnosis was retro-orbital haematoma along with intracranial haemorrhage. Orbital injury was also suspected and a B-scan was planned.

## Imaging findings

**Figure 1.**
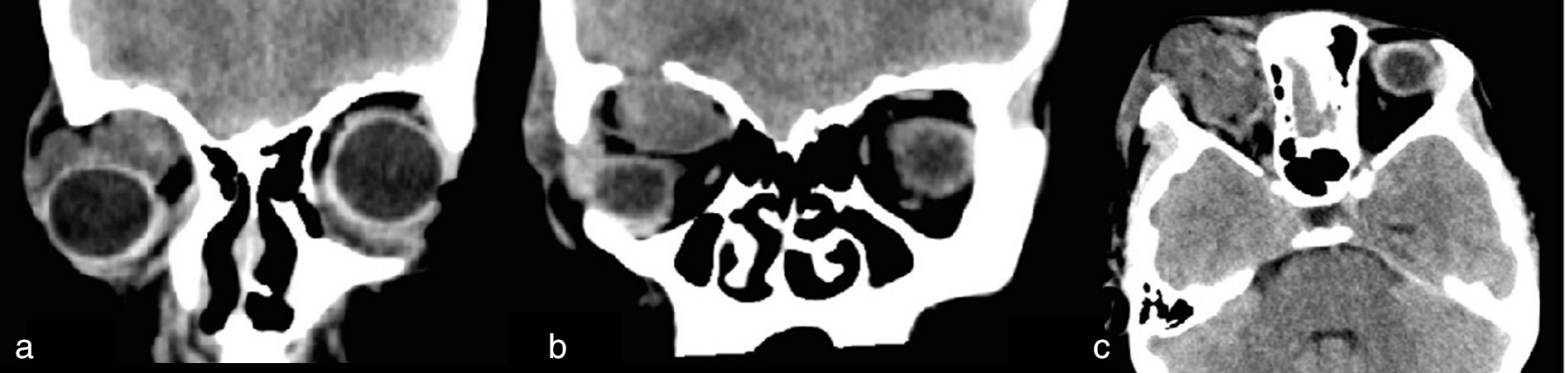
Coronal reformatted (a, b) and axial (c) CT soft tissue window images showing presence of a heterogeneous soft tissue density lesion in the right orbit protruding from a bone defect in the orbital roof. The thin dura is seen limiting the caudal extension of the herniated brain parenchyma.

**Figure 2.**
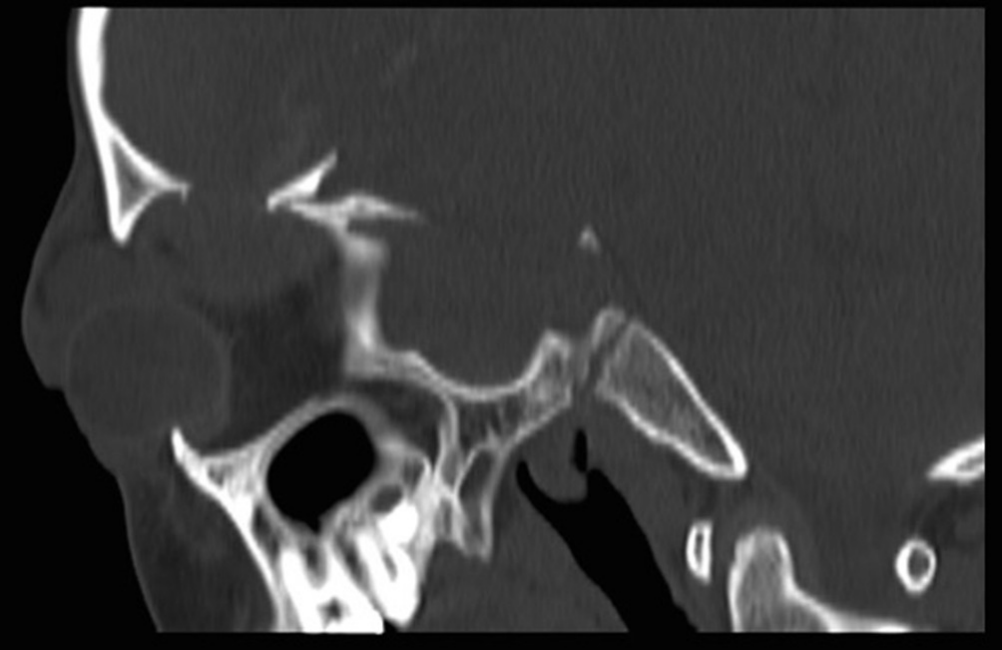
Sagittal reformatted CT bone window image showing the superoposteriorly displaced bone fragment.

**Figure 3.**
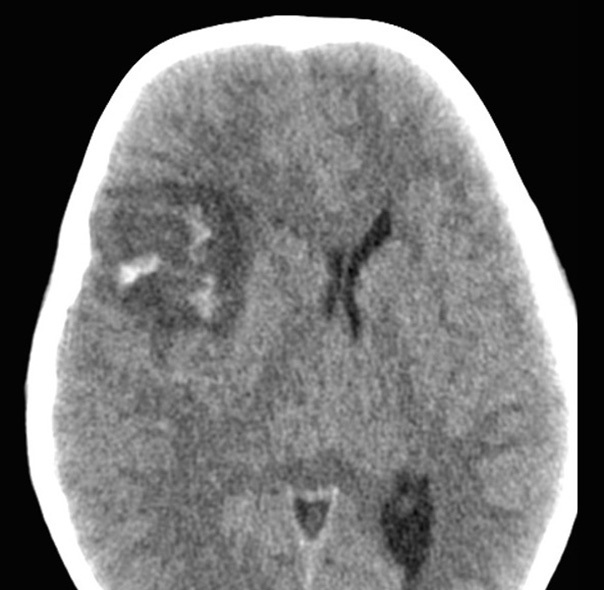
Axial CT soft tissue image showing a contusion injury in right frontal brain parenchyma and midline shift towards the contralateral side.

**Figure 4.**
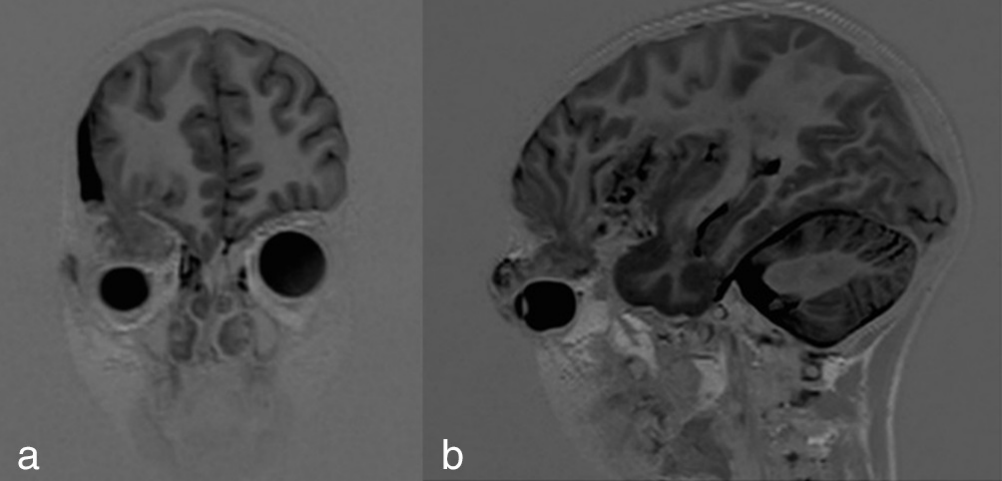
(a, b) Coronal and sagittal thin section *T*_1_ weighted MRI showing herniation of the brain tissue from the cranial cavity into the right orbit.

**Figure 5.**
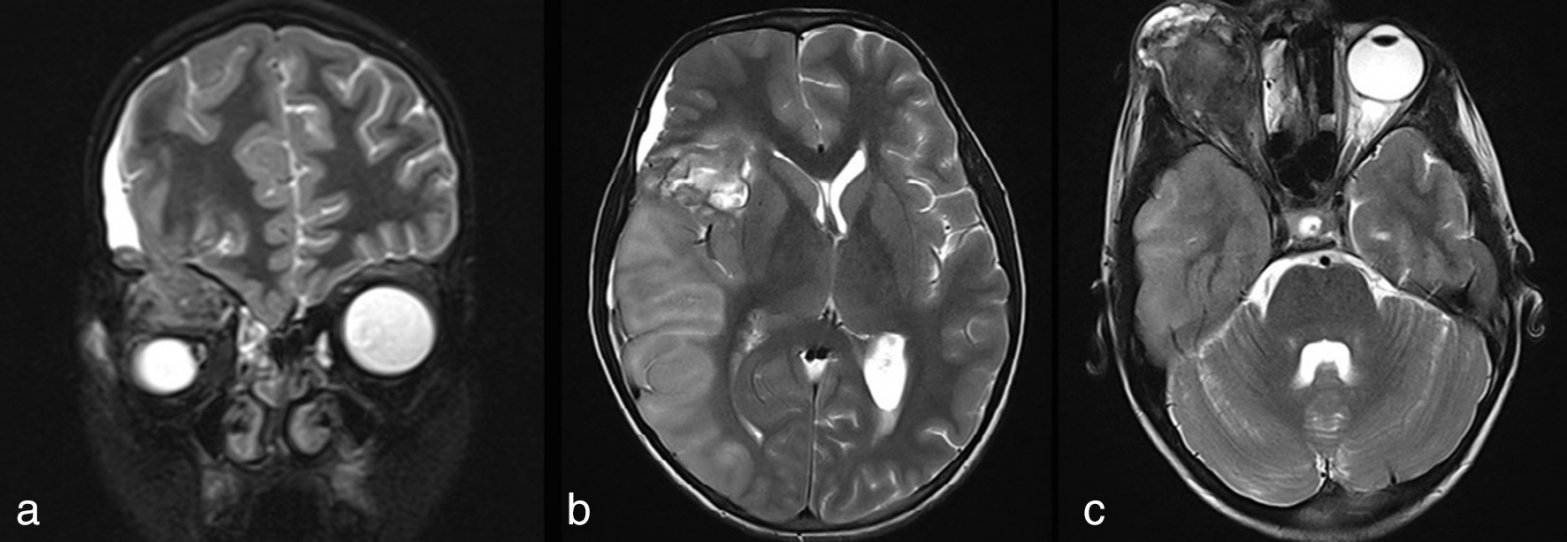
Coronal thin section (a) and axial and sagittal (b, c) *T*_2_ weighted MRI showing herniation of the brain tissue from the cranial cavity into the right orbit. Contusion injury is seen in the right frontal lobe with associated oedema and midline shift towards the contralateral side. Proptosis is seen in the axial image.

The CT scans revealed a fracture of the roof of the right orbit with posterosuperior displacement of the bone fragment. A hypodense, heterogeneous lesion was seen extending from the anterior cranial fossa into the right orbit, causing anterior protrusion of the eyeball that resulted in proptosis (Figures 1 and 2). Inferiorly, the lesion was limited by a thin membranous structure, which was suspected to be the dura. The lesion was suspected to be a herniation of a part of the frontal lobe, and haematoma was considered as a differential diagnosis. A small area of the haematoma with surrounding oedema and associated midline shift towards the left side was noted (Figure 3). MRI was advised for confirmation of the diagnosis. MRI of the brain, including the orbits, performed on a 3-T scanner confirmed herniation of the basal aspect of the right frontal lobe and other findings previously seen on the CT study (Figures 4 and 5).

## Treatment

The periorbital oedema and blackening of the eyelid were managed conservatively. The patient’s vision recovered completely since the eye swelling and proptosis decreased, although proptosis was not completely reduced. The vomiting stopped and the patient’s Glasgow Coma Scale score became normal.

## Management/outcome, follow-up and discussion

The patient recovered from most of his symptoms, except proptosis. The problem was to the patient and his parents and surgical correction was advised for which they were referred to a high complexity healthcare.

Although orbital encephaloceles are more common in the younger population, their occurrence in adults has also been reported.^1^ Proptosis, eye swelling, visual disturbances, subconjunctival haemorrhage and restricted movement of the eye are the most common presenting symptoms, with compression leading to optic nerve atrophy being the main long-term complication.^5,6^ Oedema of the retro-ocular tissues results in congestion of the veins, which pushes the eyeball forward. This leads to stretching of the extraocular muscles and the nerves, which further compresses the draining veins.^7^ Beyond a certain limit, the retinal artery gets occluded, resulting in permanent visual loss.

Isolated fractures of the orbit are classified as blow-in or blow-out fractures according to the displacement of the fractured bony fragment. If the fractured fragment lies within the orbit, it is classified as a blow-in fracture, and if the fractured fragment lies outside the orbit in the periorbital space, it is classified as a blow-out fracture. A blow-in fracture results in increased intraorbital pressure and/or impingement syndromes, which sometimes leads to irreversible neurological damage, whereas a blow-out fracture causes disruption of the orbital wall (usually inferior or medial), resulting in entrapment syndromes of the periorbital structures.^3^ Growing fractures of the orbit are associated with orbital trauma.^5^

Post-traumatic cases requiring urgent orbital surgery include: progressive loss of vision, progressive proptosis and radiologically demonstrated bony spicule pressing on the optic nerve. Visual disturbances along with increased intraorbital pressure are the two most common causes of emergency surgery in cases of orbital trauma.^3,5^ Materials used in reconstruction of the orbital roof include titanium mesh and screws with a mixture of bone powder and fibrin glue, and temporalis fascia as grafts. The former supposedly has better cosmetic results.^3,8^ Surgeries are sometimes delayed by 7–10 days until the swelling subsides.^9^

Previous case reports have focused on the clinical and management aspects of orbital encephalocele. Not much emphasis has been placed on imaging findings of the entity. Imaging is frequently required in orbital trauma as clinical evaluation is usually limited by soft tissue swelling. Plain film radiographs do not provide adequate information regarding the extent of injury, hence CT scan is the investigation of choice.^10^ CT scan has proven to be an extremely reliable investigation in cases of orbital trauma, more so with fractures of the roof of the orbit, as was in our case.^10^

## Learning points

1. The diagnosis of orbital encephalocele would surely have been missed without optimum imaging investigation, and we here would like to emphasize the importance of cross-sectional imaging in cases of orbital trauma.
2. Although imaging with CT is highly recommended and is the imaging of choice in cases of trauma, MRI is the imaging investigation of choice for orbital encephalocele.

## Consent

Written informed consent was taken from the patient's parents.
